# BTLA-Expressing Dendritic Cells in Patients With Tuberculosis Exhibit Reduced Production of IL-12/IFN-α and Increased Production of IL-4 and TGF-β, Favoring Th2 and Foxp3^+^ Treg Polarization

**DOI:** 10.3389/fimmu.2020.00518

**Published:** 2020-03-31

**Authors:** Jun-Ai Zhang, Yuan-Bin Lu, Wan-Dang Wang, Gan-Bin Liu, Chen Chen, Ling Shen, Hou-Long Luo, Huan Xu, Ying Peng, Hong Luo, Gui-Xian Huang, Du-Du Wu, Bi-Ying Zheng, Lai-Long Yi, Zheng W. Chen, Jun-Fa Xu

**Affiliations:** ^1^Department of Clinical Immunology, Institute of Laboratory Medicine, Guangdong Medical University, Dongguan, China; ^2^Guangdong Provincial Key Laboratory of Medical Molecular Diagnostics, Guangdong Medical University, Dongguan, China; ^3^Department of Clinical Medicine Laboratory, Affiliated Xiaolan Hospital, Southern Medical University, Zhongshan, China; ^4^Department of Respiration, Dongguan 6th Hospital, Dongguan, China; ^5^Department of Microbiology and Immunology, Center for Primate Biomedical Research, University of Illinois College of Medicine, Chicago, IL, United States; ^6^School of Pharmacy, Guangdong Medical University, Dongguan, China

**Keywords:** tuberculosis, B and T lymphocyte attenuator, dendritic cells, IL-12, IFN-α, CD4^+^ T cell

## Abstract

Little is known about how tuberculosis (TB) impairs dendritic cell (DC) function and anti-TB immune responses. We previously showed that the B and T lymphocyte attenuator (BTLA), an immune inhibitory receptor, is involved in TB pathogenesis. Here, we examined whether BTLA expression in TB affects phenotypic and functional aspects of DCs. Active TB patients exhibited higher expression of BTLA in myeloid dendritic cells (mDCs) and plasmacytoid DCs (pDCs) subsets compared with healthy controls (HCs). BTLA expression was similarly high in untreated TB, TB relapse, and sputum-bacillus positive TB, but anti-TB therapy reduced TB-driven increases in frequencies of BTLA^+^ DCs. BTLA^+^ DCs in active TB showed decreased expression of the DC maturation marker CD83, with an increased expression of CCR7 in mDCs. BTLA^+^ DCs in active TB displayed a decreased ability to express HLA-DR and to uptake foreign antigen, with a reduced expression of the co-stimulatory molecule CD80, but not CD86. Functionally, BTLA^+^ DCs in active TB showed a decreased production of IL-12 and IFN-α as well as a reduced ability to stimulate allogeneic T-cell proliferative responses. BTLA^+^ mDCs produced larger amounts of IL-4 and TGF-β than BTLA^−^ mDCs in both HCs and APT patients. BTLA^+^ DCs from active TB patients showed a reduced ability to stimulate Mtb antigen-driven Th17 and Th22 polarizations as compared to those from HCs. Conversely, these BTLA^+^ DCs more readily promoted the differentiation of T regulatory cells (Treg) and Th2 than those from HCs. These findings suggest that TB-driven BTLA expression in DCs impairs the expression of functional DC surrogate markers and suppress the ability of DCs to induce anti-TB Th17 and Th22 response while promoting Th2 and Foxp3^+^ Tregs.

## Summary

B and T lymphocyte attenuator (BTLA), an inhibitory receptor, is widely expressed on hematopoietic and non-hematopoietic cells. BTLA interacts with herpes virus entry mediator (HVEM) to favor the induction of Treg cell differentiation, the inhibition of T and B cell proliferation, and the production of cytokines. We previously showed that BTLA was involved in TB pathogenesis. Here, we found that active TB patients exhibited higher expression of BTLA in mDC and pDC subsets than healthy controls (HCs). BTLA^+^ DCs in active TB showed decreased expression of the DC maturation marker CD83, decreased ability to express HLA-DR and to uptake foreign antigen, and reduced expression of the co-stimulatory molecule CD80, but not CD86. BTLA^+^DCs in active TB showed decreased production of IL-12 and IFN-α and increased production of IL-4 and TGF-β, as well as a reduced ability to stimulate allogeneic T-cell proliferative responses. Notably, BTLA^+^ DCs from active TB patients showed a reduced ability to stimulate Mtb antigen-driven Th17 and Th22 polarizations, but favor Th2 and Foxp3^+^ Treg differentiation. These findings suggest that TB-driven BTLA expression is associated with an increased production of IL-4 and TGF-β by DCs and with decreased abilities to produce the key cytokine IL-12, and to induce T cell proliferation and differentiation into Th subsets.

## Introduction

Tuberculosis (TB) remains a leading cause of mortality among infectious diseases, with 10.4 million new cases (including 600,000 new cases with resistance to rifampicin, the most potent first-line drug) and 1.7 million deaths in 2016 (1). Despite extensive studies, little is known about the immune mechanisms involved in TB infection and progression, since the different hosts respond in three different ways: complete clearance, latent infection, or chronic TB after *Mycobacterium tuberculosis* (Mtb) exposure. In fact, one-third of the world population is estimated to be latently infected with Mtb, but only <10% of the infected individuals would eventually develop the disease. The persistence of Mtb in discrete lesions in healthy individuals indicates that although the immune system can effectively constrain the pathogen, it fails to eradicate the infection (2, 3). The chronic nature of this infection implies that Mtb has developed strategies to avoid clearance by the innate and adaptive immune responses (4, 5).

Dendritic cells (DC) are the major antigen-presenting cells (APC) in the immune system and play a critical role in adaptive immunity by activating naïve T cells, maintaining tolerance to self-antigens, and bridging the innate and adaptive responses (6). The DC family comprises of phenotypically and functionally specialized subsets such as myeloid dendritic cells (mDCs) and plasmacytoid DCs (pDCs). The mDCs express CD11c, require granulocyte-macrophage colony-stimulating factor (GM-CSF) for growth, survival, and antigen uptake, and play roles in T cell activation and secretion of interleukin (IL)-12 and IL-18. The pDCs express CD123, are dependent on IL-3 for survival and produce high levels of interferon (IFN)-α in response to viral infection (7, 8). The DCs sense the pathogen-associated molecular patterns (PAMPs) of TB bacilli with the aid of innate receptors such as TLRs and RLRs (9, 10). Interestingly, immature DCs explore the immunological milieu of the tissue in which they reside. Upon activation, immature DCs undergo a transformation process that includes up-regulation of class I and class II MHC molecules and co-stimulatory molecules (such as CD80 and CD86), production of IFNs and pro-inflammatory cytokines (IL-12, IL-15, IL-18, and IL-10), and radical changes in the chemokine receptor and adhesion molecule profiles (9, 11–13). The activated mature DCs migrate to the lymphoid organs, where they interact with and stimulate both naïve and primed T cells (11, 12).

It is suggested that DCs play a pivotal role in immune responses to TB (14). In fact, we recently demonstrated that the absolute number of total DCs (tDCs), mDCs, and pDCs in individuals with active pulmonary tuberculosis (APT) was decreased compared with healthy controls (HCs); this decrease was associated with prolonged and complicated TB, TB burden, and over-reactive inflammation (15). There was also an altered expression of the maturation surface markers (CD83 and CCR7), the antigen presenting molecule HLA-DR, and the co-stimulatory molecules (CD80 and CD86) of DCs in APT patients (15). These data suggest that Mtb might modulate the DC functions during induction or development of TB, but further studies are needed to define the TB-induced changes in DC function and to understand the underlying mechanism.

The B and T lymphocyte attenuator (BTLA) is an inhibitory receptor and shares structural and functional similarities with cytotoxic T-lymphocyte antigen-4 (CTLA-4) and programmed death-1 (PD-1) (16). BTLA is widely expressed on hematopoietic and non-hematopoietic cells such as T and B lymphocytes, DCs, natural killer (NK) cells, and other cells in the lymphoid compartment. BTLA interacts with the herpes virus entry mediator (HVEM; TNFRSF14), a TNFR super-family member found on T, B, NK, DCs, and myeloid cells (17). The ligation of BTLA with HVEM is shown to favor the induction of Treg cell differentiation, the inhibition of T and B cell proliferation, and the production of cytokines (18–20). As a negative immune regulator, BTLA is crucial not only for the maintenance of peripheral tolerance but also for the pathogenesis of inflammatory disorders, autoimmune diseases, cancer, and infectious disease (17, 20–23). In a previous study, we demonstrated that BTLA expression was involved in effector memory functions for αβT cells in patients with active TB (24) and associated with increased Foxp3 expression in CD4^+^ T cells in dextran sulfate sodium-induced colitis (25). Moreover, we found that BTLA is highly expressed in CD11c-expressing APCs, and is associated with decreased capacity to stimulate allogeneic T cell proliferation (26).

Whether BTLA expression plays a role in the function of DC is still unknown. In the present study, we examined the expression of BTLA in DCs in patients with active TB and investigated whether BTLA expression could affect the stimulatory capacity of DCs on T cell proliferation and polarization.

## Materials and Methods

### Subjects

A total of 73 patients (14–69 years of age) with sputum smears positive for Mtb and eligible for first-line treatment were diagnosed with APT based on clinical symptoms, chest X radiography, and acid-fast bacilli (AFB) staining of sputum smears, positive bacterial culture, bronchoalveolar lavage (BAL), or direct biopsy at the Dongguan Sixth People's Hospital (Dongguan, China). These TB patients received individualized treatment with anti-tuberculosis drugs (ATDs) such as isoniazid, rifampicin, pyrazinamide, and ethambutol. The tests were repeated during and after treatment to monitor treatment response. A total of 35 volunteers (15–60 years of age) were recruited as healthy controls (HC). They had to be negative for any bacteriological and clinical evidence of TB. Subjects with HIV infection, diabetes, cancer, autoimmune diseases, and immunosuppressive treatment history were excluded from the study. All blood samples were collected before or within 1 week after the patients were administered ATDs. Tables S1, S2 present the characteristics of the subjects.

### Peripheral Blood Mononuclear Cells Preparation

Peripheral blood mononuclear cells (PBMCs) were prepared as described previously (24, 26). Blood samples (10 mL) were drawn from each subject in acid citrate dextrose (ACD)-containing blood collection tubes. PBMCs were freshly isolated from blood by standard Ficoll (GE Healthcare) density gradient centrifugation. Cell viability was determined by trypan blue exclusion (>95% in all experiments).

### Antibodies for Flow Cytometry

The following mouse anti-human antibodies were used for flow cytometry and cell sorting: CD11c-APC-eFluor780 (BU15, eBioscience), CD11c-PE-Cy7 (B-ly6, BD Pharmingen), Lin1-FITC (SK7, 3G8, SJ25C1, L27, eMfP9, NCAM16.2, BD Biosciences), Lin1-APC (UCHT1, HCD14, 3G8, HIB19, 2H7, HCD56, BioLegend), CD123-PerCP-CyTM5.5 (7G3, BD Biosciences), HLA-DR-APC-eFluor780 (LN3, eBioscience), HLA-DR-APC (G46-6, BD), CD86-FITC (FUN-1, BD Biosciences), CD80-FITC (2D10.4, eBioscience), CD83-FITC (HB15e, BD Biosciences), CCR7-FITC (150503, BD Biosciences), BTLA-APC (MIH26, Biolegend), BTLA-PE (J168-540, BD Pharmingen), CD3-PerCp-Cy5.5 (SP34-2, BD Biosciences), CD4-PerCP-Cy5.5 (RPA-T4, eBioscience), CD4-APC (L200, BD Biosciences), CD27-PE-Cy7 (0323, eBioscience), CD45RA-FITC (HI100, Biolegend), CD27-PE-Cy7 (0323, eBioscience), CD45RA-FITC (HI100, Biolegend), Foxp3-PE (PCH101, eBioscience), IFN-α-FITC (MMHA-11, InterferonSource), IL-12-FITC (MHCIL1201, Invitrogen), IL-4-FITC (MP4-25D2, Biolegend), and IFN-γ-APC-eFluor780 (45.B3, eBioscience).

### Flow Cytometry Analysis

The expression of BTLA, DC maturation markers CD83 and CCR7, DC antigen presenting molecules HLA-DR, and co-stimulatory molecules CD80 and CD86 on tDCs and DC subsets was determined by flow cytometry (15, 26, 27). Heparin sodium-anticoagulated whole blood (100 μL) was probed with antibodies for 30 min, followed by lysis of red blood cells. After washing, the stained cells were resuspended in 200 μL of 2% FBS-PBS containing 2% paraformaldehyde, and the samples were analyzed using a BD FACS-Canto II flow cytometer and the Sunglow software (Tree Star).

The gating strategy is shown in Figure S1. For surface marker analysis, PBMCs were gated on FSC and SSC dot plots. Total DCs were gated from PBMCs by HLA-DR expression and Lin1 negativity. The DC subsets were gated as: CD11c^+^CD123^−^ for mDCs and CD11c^−^CD123^+^ for pDCs. The expression of BTLA, DC maturation markers CD83 and CCR7, co-stimulatory molecules CD80 and CD86, and antigen-presenting molecule HLA-DR was analyzed in mDCs and pDCs.

For cell sorting of naïve T cells, PBMCs were stained with CD3-PerCp-Cy5.5 (SP34-2, BD Biosciences), CD27-PE-Cy7 (0323, eBioscience), and CD45RA-FITC (HI100, Biolegend). Naïve T cells were gated first on FSC and SSC dot plots for lymphocyte, and then gated by CD3^+^CD45RA^+^CD27^+^. For cell sorting of BTLA^+^ DCs (BTLA^+^HLA-DR^+^LIN1^−^) or BTLA^−^ DCs (BTLA^−^HLA-DR^+^LIN1^−^), PBMCs were gated on FSC and SSC dot plots. BTLA^+^ DCs were gated by the surface markers BTLA^+^ HLA-DR^+^ LIN1^−^, while BTLA^−^ DCs were gated by the surface markers BTLA^−^ HLA-DR^+^ LIN1^−^.

### mDC and pDC Intracellular Cytokine Staining

Intracellular cytokine staining (ICS) was performed to detect the cytokine secretion by the DCs, as described previously (26–28). PBMCs (1 × 10^6^) in round-bottom 96-well plates were used to measure IL-4, IL-6, and TGF-β in mDCs. The DCs were incubated for 6 h at 37°C with 5% CO_2_. Some cells were pre-stimulated with H37Rv lysate (100 μg/mL) for 3 days for testing the production of IFN-α in pDCs. The cells were transferred from the 96-well plates into 5-mL polystyrene round-bottom tubes (BD Biosciences) for the detection of surface markers (using the Lineage Cocktail-FITC (348801, Biolegend), CD11c-PE (6137918, BD Biosciences), HLA-DR-PerCP (6054701, BD Biosciences), and BTLA-APC (344510, Biolegend) for IL-4 and IL-6 detection; and the Lineage Cocktail-APC (348803, Biolegend), CD11c-PE-Cy7 (301608, Biolegend), HLA-DR-PerCP (6054701, BD Biosciences), and BTLA-PE (558485, BD Biosciences) for TGF-β detection), and of intracellular cytokines [IL-6-PE-Cy7 (501120, Biolegend), IL-4-APC-Cy7 (500834, Biolegend), and TGF-β-FITC (349606, Biolegend)]. The cells were washed once with 2% FBS-PBS and stained at room temperature for at least 25 min with the surface marker antibodies. For ICS, PBMCs were further washed with 2% FBS-PBS and permeabilized with the Fixation/Permeabilization Kit with GolgiPlug (555028, BD Biosciences) for 30 min at 4°C, and then stained for 25 min with IL-4, IL-6, and TGF-β antibodies, followed by washes with 2% FBS-PBS buffer. The cells were resuspended in 2% formaldehyde-PBS and subjected to flow cytometry analysis. Matched isotype IgG were used as a negative control for cytokines or surface markers.

### CFSE Proliferation Assay for the Stimulatory Function of DCs to Allogeneic T Cells

Mixed leukocyte culture (MLC) was used to test the capacity of DCs while stimulating the allogeneic T cell proliferation (26). BTLA^+^ DCs and BTLA^−^ DCs were sorted by flow cytometry. PBMCs were isolated from eight active TB patients with a positive sputum smear and from eight volunteers, and stained with mouse anti-human antibody-fluorochrome cocktails, including Lin1 (BU15; eBioscience), mouse anti-human HLA-DR, CD11c, and CD123, BTLA-APC (MIH26; Biolegend), and BTLA-PE (J168-540, BD Pharmingen) monoclonal antibodies, followed by sorting on a FACS Aria II. CD3^+^ T cells were isolated from an allogenic donor using a human T cell isolation kit, according to the manufacturer's instructions (StemCell Biotech, Vancouver, Canada). Isolated cells, with >97% purity, were used for subsequent experiments. To evaluate the T cell proliferation, some of the purified CD3^+^ T cells were labeled with 20 μM CFSE (Beyotime), and 5 × 10^4^ cells/well were cultured with sorted BTLA^+^DCs or BTLA^−^DCs (5 × 10^3^ cells/well) in 96-well round-bottom plates for 7 days. T cells alone served as the negative control. The cells were harvested, stained with CD3-PerCp-Cy5.5 (BD Pharmingen) and CD4-APC (BD Biosciences) for 30 min at 4°C, acquired using a BD FACS-Canto II flow cytometer, and analyzed using the FlowJo software. The CFSE-low cells were quantified as a percentage of proliferating cells.

### FITC-Dextran Uptake Assay

The FITC-dextran uptake assay was performed by incubating the cells with FITC-dextran in duplicate plates at 4 or 37°C, as described previously (26, 29). PBMCs (1 × 10^6^ in 200 μL) were incubated with 1 mg/mL FITC-dextran in 5-mL Falcon™ polystyrene round-bottom tubes (BD Biosciences). The FITC-dextran solution was vortexed for 30 s before use. One plate was incubated at 37°C and the other at 4°C for 1 h to determine the baseline FITC-dextran uptake level. Then, the cells were washed twice with cold 2% FBS-PBS and stained with mouse anti-human antibody-fluorochrome cocktails, including Lin1-APC, mouse anti-human HLA-DR-PE-Cy7, CD11c-APC-eFluor780, CD123-PerCP-CyTM5.5, and BTLA-PE at 4°C for 30 min. The cells were resuspended in 200 μL of 2% FBS-PBS containing 2% paraformaldehyde and analyzed on a BD FACS-Canto II flow cytometer. The percentage of phagocytosis was determined as following: Percentage of phagocytosis of DCs (%) = Percentage of phagocytosis of DCs at 37°C (%)- Percentage of phagocytosis of DCs at 4°C (%).

### Stimulatory Capacity of DCs on Naïve T Cell Polarization

Naïve T cells (CD45RA^+^CD27^+^) and BTLA^+^ DCs (BTLA^+^HLA-DR^+^LIN1^−^) or BTLA^−^ DCs (BTLA^−^HLA-DR^+^LIN1^−^) were sorted by Beckman MoFlo XDP flow cytometer from the PBMCs from six patients with active TB and five HCs. PBMCs from APT patients or HCs were stained with each surface marker antibody. Naïve T cells and BTLA^+^ DCs or BTLA- DCs were sorted by respective surface markers by flower cytometry and the purity of naïve T cells and DCs was determined by flow cytometry. Naïve T cells (10^5^ cells/well) were cultured in the presence of sorted BTLA^+^ DCs or BTLA^−^ DCs (10^4^ cells/well) in 96-well round-bottom plates for 5 days and stimulated with 100 μg/mL H37Rv lysate. Subsequently, the cells were harvested by centrifugation at 1,500 rpm for 5 min, and the supernatant frozen in cryogenic refrigerators. The cells were stained with membrane surface antibody CD4-PerCP-Cy5.5 and intracellular antibodies such as, IL-4-FITC, Foxp3-PE, IFN-γ-APC-eFluor780, IL-17A-PE-Cy7, and IL-22-APC. Finally, the polarization of T cells was analyzed by BD FACS Verse flow cytometer.

### Statistical Analysis

The Kolmogorov-Smirnov normality test was performed to determine whether the dataset followed the normal distribution. The Student's *t*-test was used for the comparison of two groups. ANOVA with the Bonferroni (equal variances) or Dunnett (unequal variances) was used for the comparison of multiple groups. All statistical analyses were performed using GraphPad Prism version 5.0 (GraphPad Software Inc.) or SPSS 22.0 for Windows (IBM Corp.). In all cases, two-sided *P* < 0.05 were considered statistically significant.

## Results

### Active TB Patients Exhibit High Expression of BTLA in mDCs and pDCs

**Figure 1:**
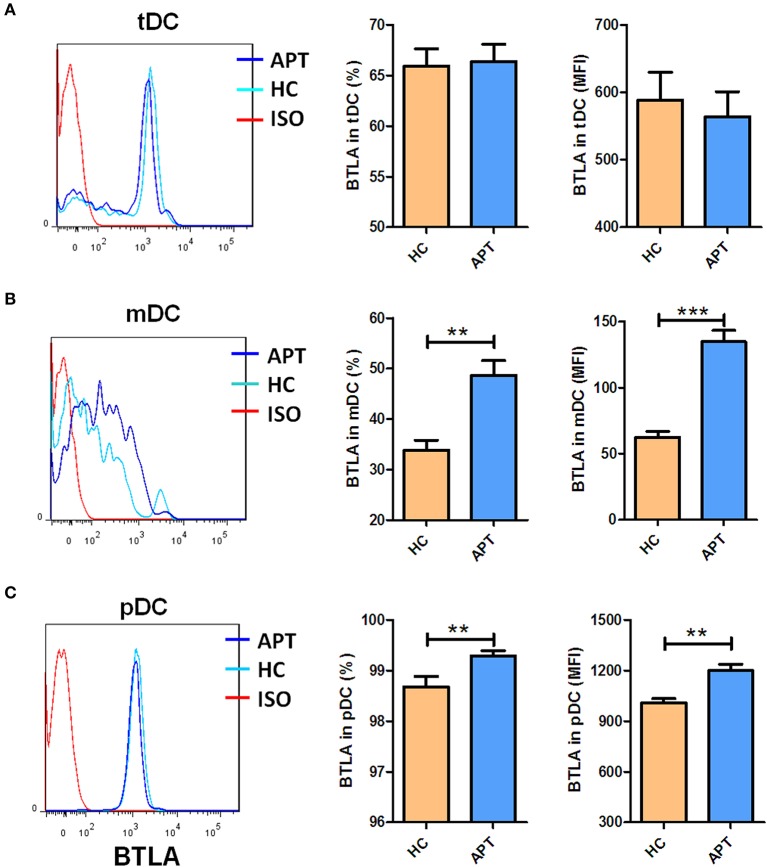
Active TB patients exhibited higher expression of BTLA in mDC and pDC subsets than HCs. PBMCs were prepared from patients with APT (*n* = 73) and HCs (*n* = 35). Cells were assessed for frequencies of BTLA-expressing DC subsets by flow cytometry. The gating strategy is shown in Figure S1. The left columns of **(A–C)** show the representative histograms for flow cytometry analysis of the expression of BTLA in tDCs **(A)** and its subsets mDCs **(B)**, and pDCs **(C)**. The middle columns of **(A–C)** show the mean frequencies of BTLA-expressing cells in tDCs **(A)** and its subsets mDCs **(B)** and pDCs **(C)**. The right columns of **(A–C)** show the mean fluorescence intensity (MFI) of BTLA expression in tDCs **(A)** and its subsets mDCs **(B)** and pDCs **(C)**. The P-value is shown in each column (Student *t*-test). ^**^P < 0.01, ^***^P < 0.001.

BTLA expression in DCs in patients with APT has not been reported, although we previously found that BTLA was highly expressed in CD11c^+^ APCs in active TB patients (26). Here, we used flow cytometry to examine the frequencies of BTLA-expressing DC subsets from TB patients and HCs. Representative flow cytometry data are shown in Figure 1. tDCs from TB patients and HCs showed a lack of differences in BTLA-expression and BTLA mean fluorescence intensity (MFI) (Figure 1A). APT patients showed a much higher percentage of BTLA-expressing mDCs than that in HCs (Figure 1B). Consistently, the BTLA MFI in mDCs from APT patients was also much greater than that in HCs. APT patients exhibited significantly higher frequencies of BTLA-expressing pDCs with greater BLTA MFI in PBMCs than did HCs (Figure 1C, lower panel). These results suggest that active TB drives higher BTLA expression in DC subsets, especially in pDCs. These results are consistent with our previous observation of the high expression of BTLA in CD11c-expressing APCs in TB (26).

### BTLA Expression According to Mtb Infection Patterns and After Anti-TB Therapy

**Figure 2:**
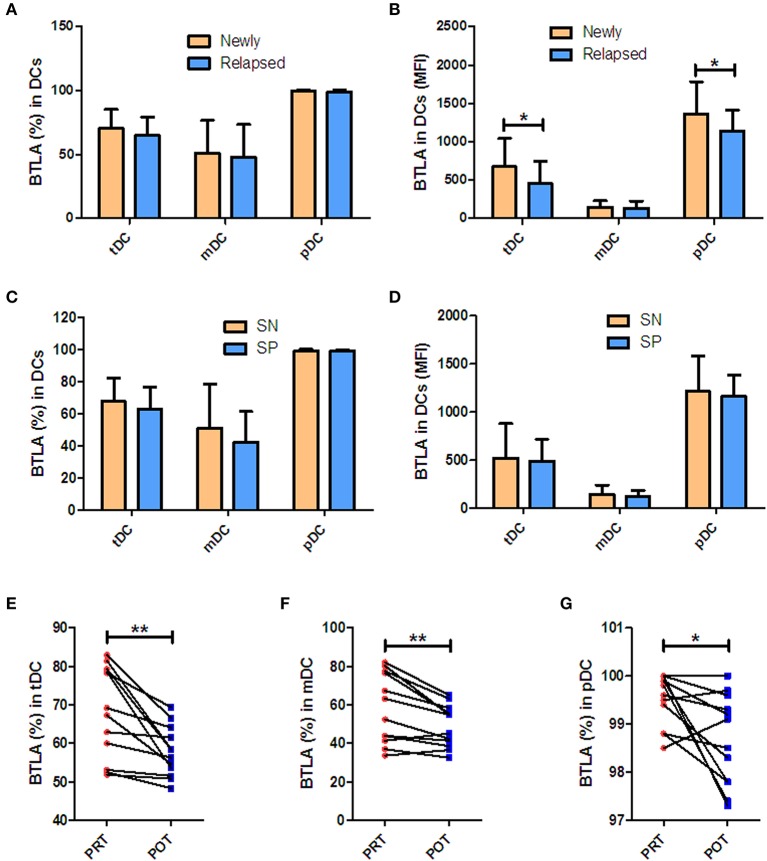
BTLA expression in Mtb infection patterns and after anti-TB therapy. **(A,C)** show percentages of BTLA^+^DCs in untreated TB (newly) vs. TB relapse (relapsed), and sputum-positive (SP) vs. sputum-negative (SN) patients, respectively. **(B,D)** show the MFI of BTLA on DCs in new vs. relapsed TB and SP vs. SN patients, respectively. **(E–G)** show the decrease in BTLA^+^ tDCs **(E)**, mDCs **(F)**, and pDCs **(G)** in PRT vs. POT of the new APT patients (*n* = 10). ^*^P < 0.05, ^**^P < 0.01 (Student *t*-test).

Next, we sought to determine if increased BTLA expression in DC subsets in active TB patients were linked to different clinical patterns of TB disease. We compared frequencies of BTLA^+^ DCs between untreated TB, TB relapse, and TB positive in sputum vs. negative. Overall, all these patterns of TB patients exhibited similar high frequencies of BTLA^+^ DCs (Figures 2A–D), although BTLA MFI in tDCs and pDCs was lower in relapsed TB and other patterns of TB (Figure 2B). Interestingly, effective anti-TB therapy reduced the frequencies of BTLA^+^ tDCs, mDCs, and pDCs in TB patients (Figures 2E–G). These results provide additional data suggesting that TB drives high expression of BTLA in DCs.

### BTLA^+^ DCs in Active TB Shows a Decreased Expression of CD83, With an Increased Expression of CCR7 in mDCs

**Figure 3:**
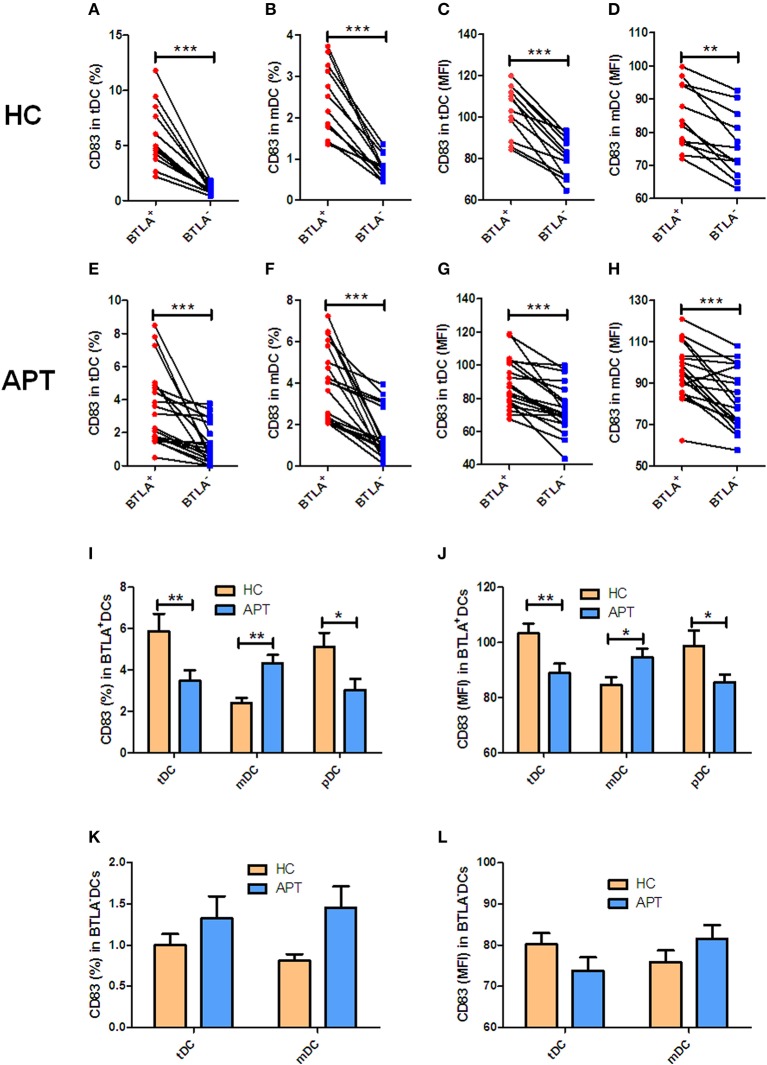
BTLA^+^DCs in active TB showed a decreased expression of the DC maturation marker CD83. The expression of the maturation surface marker CD83 on DCs was determined by flow cytometry (*n* = 12). The gating strategy is shown in Figure S1. **(A,B)** show the positive rate (%) of CD83 expression between BTLA^+^ DCs and BTLA^−^ DCs of HCs. **(C,D)** show the MFI of CD83 expression between BTLA^+^ DCs and BTLA^−^ DCs in HCs. **(E,F)** show the positive rate (%) of CD83 expression between BTLA^+^ DCs and BTLA^−^ DCs in APT patients. **(G,H)** show the MFI of CD83 expression between BTLA^+^ DCs and BTLA^−^ DCs in APT patients. **(I,J)** show the positive rate (%) **(I)** and MFI **(J)** of CD83 expression in BTLA^+^ tDCs/mDCs/pDCs between HCs and APT patients. **(K,L)** show the positive rate (%) **(I)** and MFI **(K)** of CD83 expression in BTLA^−^ tDCs and mDCs between HCs and APT patients. The *P*-value is shown in each column (Student *t*-test). ^*^P < 0.05, ^**^P < 0.01, ^***^P < 0.001.

**Figure 4:**
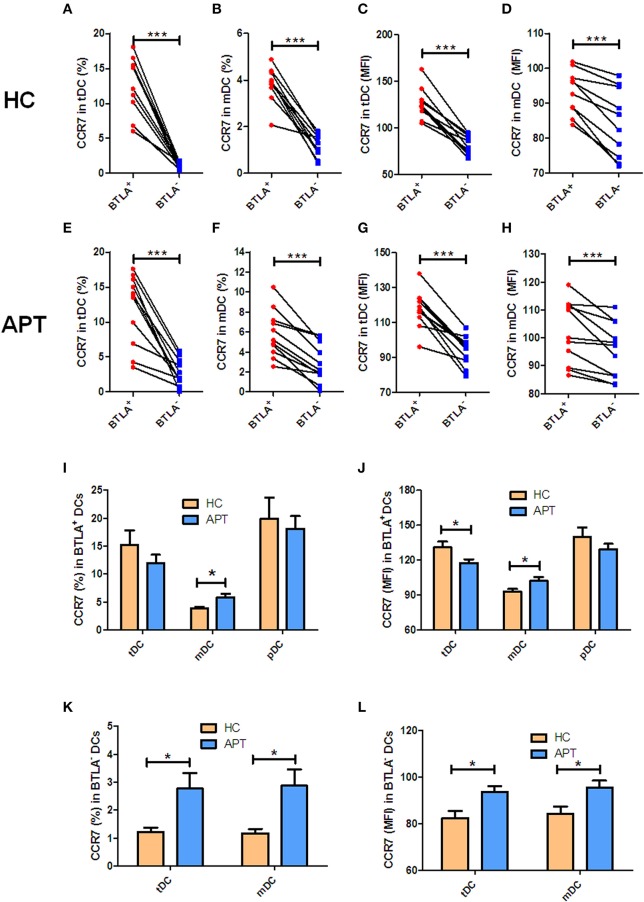
BTLA^+^ mDCs in active TB showed an increase in CCR7^+^ cell subsets. The expression of the CCR7 in DCs was determined by flow cytometry (*n* = 12). The gating strategy is shown in Figure S1. **(A,B)** show the positive rate (%) of CCR7 expression between BTLA^+^ DCs and BTLA^−^ DCs in HCs. **(C)** and **(D)** show the MFI of CCR7 expression between BTLA^+^ DCs and BTLA^−^ DCs in HCs. **(E,F)** show the positive rate (%) of CCR7 expression between BTLA^+^ DCs and BTLA^−^ DCs in APT patients. **(G,H)** show the MFI of CCR7 expression between BTLA^+^ DCs and BTLA^−^ DCs in APT patients. **(I,J)** show the positive rate (%) **(I)** and MFI **(J)** of CCR7 expression in BTLA^+^ tDCs/mDCs/pDCs between HCs and APT patients. **(K,L)** show the mean frequency (%) **(K)** and MFI **(L)** of CCR7 expression in BTLA^−^ tDCs and mDCs between HCs and APT patients. The *P*-value is shown in each column (Student *t*-test). ^*^P < 0.05, ^***^P < 0.001.

Since CD83 and CCR7 are maturation makers of DCs, we sought to examine whether BTLA expression impacts the maturation of DCs. Because nearly all pDCs express BTLA, BTLA^−^ pDCs were not sufficiently available for analysis, and we focused on tDCs and mDCs. We found that frequencies of CD83^+^/CCR7^+^ DCs and the MFI of CD83 or CCR7 in BTLA^+^ tDCs and BTLA^+^ mDCs were significantly higher than those in BTLA^−^ tDCs and BTLA^−^ mDCs in HCs and APT patients, respectively (Figures 3A–H, 4A–H). When BTLA expression was assessed for correlate of altered DCs maturation, we found that BTLA^+^ tDCs and BTLA^+^ pDCs from APT patients showed significantly lower frequencies of CD83^+^ DCs and weaker MFI of CD83 expression than did those from HCs (Figures 3I,J). Similarly, BTLA^+^ mDCs from APT patients showed higher frequencies of CCR7^+^ DCs and stronger MFI than those from HCs, despite a lack of differences in BTLA^+^ pDCs (Figures 4I,J). In contrast, BTLA^−^ mDCs from APT patients exhibited higher frequencies of CCR7^+^ DCs and MFI of CCR7 than those from HCs (Figures 4K,L), despite a lack of differences of C83 expression (Figures 3K,L). These data strongly suggest that BTLA^+^ DCs in active TB shows a decreased expression of the DC maturation marker CD83, with an increased expression of CCR7 in mDCs.

### BTLA^+^ DCs in Active TB Show a Decreased Ability to Express HLA-DR and Uptake Antigens

**Figure 5:**
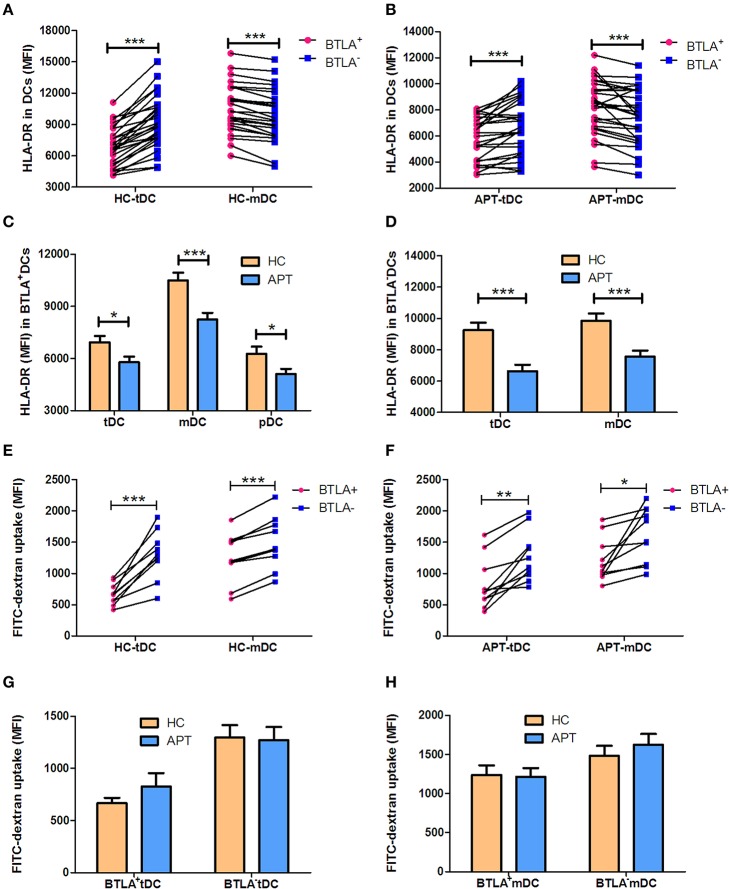
BTLA^+^ DCs in active TB showed a decreased MFI of HLA-DR, and reduced antigen uptake capacity. The MFI of the antigen presenting molecule HLA-DR in DC subsets was analyzed by flow cytometry. The antigen uptake capacity of DC subsets was evaluated by a FITC-dextran uptake test. **(A)** shows the MFI of HLA-DR expression between BTLA^+^ and BTLA^−^ tDCs and mDCs in HCs (*n* = 27), respectively. **(B)** shows the MFI of HLA-DR expression between BTLA^+^ and BTLA^−^ tDCs and mDCs in APT patients (*n* = 26). **(C)** shows the MFI of HLA-DR expression in BTLA^+^ tDCs, mDCs, and pDCs between HCs and APT patients. **(D)** Comparison of the MFI of HLA-DR expression in BTLA^−^ tDCs and mDCs between HCs and APT patients. **(E,F)** show the MFI of FITC-dextran uptake of BTLA^+^DCs and the counterpart BTLA^−^ DCs in HCs **(E)** and in APT patients **(F)**. **(G,H)** show the MFI of FITC-dextran uptake in tDCs **(G)** and mDCs **(H)** from HCs (*n* = 10) and patients with APT (*n* = 10). The *P*-values are shown in each column (Student *t*-test). ^*^P < 0.05, ^**^P < 0.01, ^***^P < 0.001.

HLA-DR, a powerful allogenic stimulator, is the MHC II molecule that can present antigen to CD4^+^ T cells. Thus, we sought to examine whether BTLA^+^ DCs showed altered expression of HLA-DR. We found that BTLA^+^ tDCs showed a lower expression of HLA-DR than BTLA^−^ tDCs in APT patients and HCs (Figures 5A,B), whereas BTLA^+^ mDCs showed a higher expression of HLA-DR than its counterpart BTLA^−^ mDCs in APT patients and HCs (Figures 5A,B). Surprisingly, all BTLA^+^ DC subsets including tDCs, mDCs, and pDCs in APT patients exhibited much lower HLA-DR expression than those in HCs (Figure 5C). In this regard, BTLA^−^ tDCs and mDCs in APT patients showed a lower HLA-DR expression than those in HCs (Figure 5D).

The reduced MHC II HLA-DR expression raises a question as to whether DCs in APT patients have a reduced capacity to uptake antigens. To address this, we conducted a FITC-dextran uptake test to examine whether BTLA^+^ DCs showed a reduced function of antigen uptake. Interestingly, BTLA^+^ tDCs and mDCs exhibited lower capacity of antigen uptake than BTLA^−^ DCs (Figures 5E,F), but no difference was seen between HCs and APT patients (Figures 5G,H). Together these results suggest that BTLA expression in DCs alters the expression of the antigen presenting molecule HLA-DR, especially APT patients, and reduces the capacity of antigen uptake.

### BTLA^+^ DCs in Active TB Show a Decreased Expression of the Co-stimulatory Molecule CD80 but Not CD86

**Figure 6:**
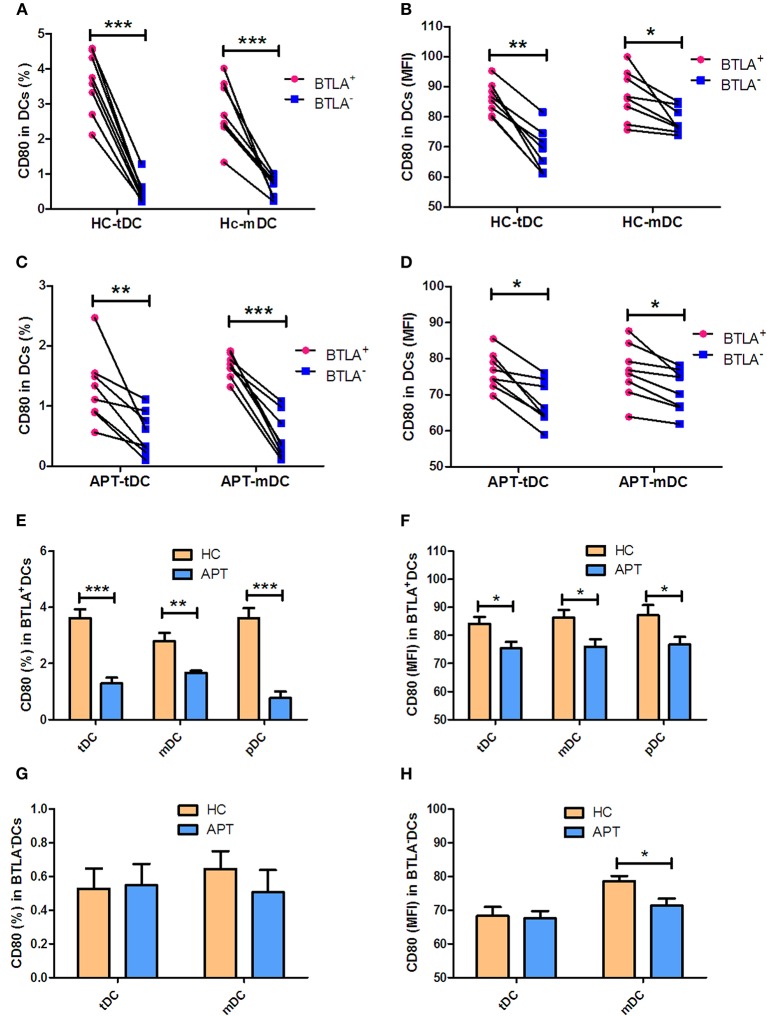
BTLA^+^ DCs in active TB showed decreased expression of the co-stimulatory molecule CD80. The expression of the co-stimulatory molecule CD80 in DCs was determined by flow cytometry (*n* = 8). The gating strategy is shown in Figure S1. **(A,B)** show the positive rate (%) **(A)** and MFI **(B)** of CD80 expression between BTLA^+^ DCs and the counterpart BTLA^−^ DCs in HCs. **(C,D)** show the positive rate (%) **(C)** and MFI **(D)** MFI of CD80 expression between BTLA^+^ DCs and BTLA^−^ DCs in APT. **(E,F)** show the mean frequency (%) **(E)** and MFI **(F)** of CD80 expression in BTLA^+^ tDCs/mDCs/pDCs between HCs and APT patients. **(G,H)** show the mean frequency (%) **(G)** and MFI **(H)** of CD80 expression in BTLA^−^ tDCs/mDCs between HCs and APT patients. The P-value is shown in each column (Student *t*-test). ^*^P < 0.05, ^**^P < 0.01, ^***^P < 0.001.

**Figure 7:**
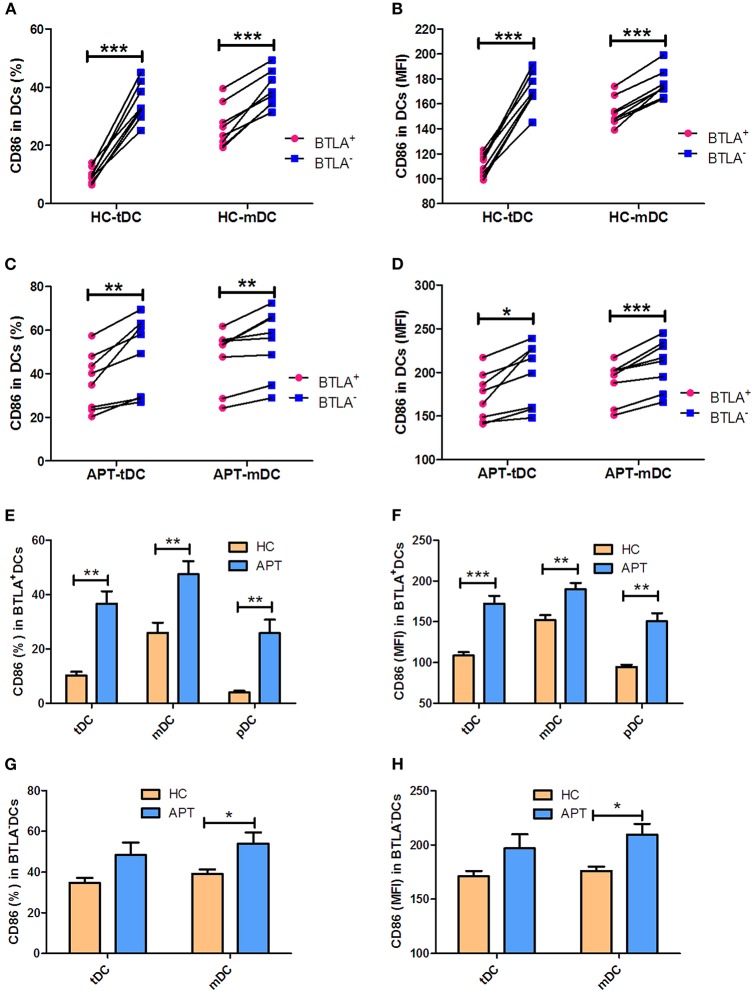
BTLA^+^ DCs in active TB showed an increased expression of the co-stimulatory molecule CD86. The expression of the co-stimulatory molecule CD86 in DCs was determined by flow cytometry (*n* = 8). The gating strategy is shown in Figure S1. **(A,B)** show the positive rate (%) **(A)** and MFI **(B)** of CD86 expression between BTLA^+^ DCs and the counterpart BTLA^−^ DCs in HCs. **(C,D)** show the positive rate (%) **(C)** and MFI **(D)** of CD86 expression between BTLA^+^ DCs and BTLA^−^ DCs in APT. **(E,F)** show the mean frequency (%) **(E)** and MFI **(H)** of CD86 expression in BTLA^+^ tDCs/mDCs/pDCs between HCs and APT patients. **(G,H)** show the mean frequency (%) **(G)** and MFI **(H)** of CD86 expression in BTLA^−^ tDCs and mDCs between HCs and APT patients. The *P*-values are shown in each column (Student *t*-test). ^*^P < 0.05, ^**^P < 0.01, ^***^P < 0.001.

The expression of the co-stimulatory molecules CD80 and CD86 in DC subsets was analyzed by flow cytometry. BTLA^+^ tDCs and mDCs displayed high expression of CD80 in both HCs (Figures 6A,B) and APT patients (Figures 6C,D). But the expression of CD86 was opposite to that of CD80 on the surface of BTLA^+^ DCs: it was highly expressed in BTLA^−^ DCs in both HCs (Figures 7A,B) and APT patients (Figures 7C,D). Intriguingly, CD80 expression in tDCs, mDCs, and pDCs was lower in APT patients than in HCs (Figures 6E–H). CD86 expression in tDCs, mDCs, and pDCs was higher in APT patients than in HCs (Figures 7E–H). These data suggest that BTLA expression in DC subsets alter the expression of co-stimulatory molecules.

### BTLA^+^ DCs in Active TB Show a Decreased Production of IL-12 and IFN-α

**Figure 8:**
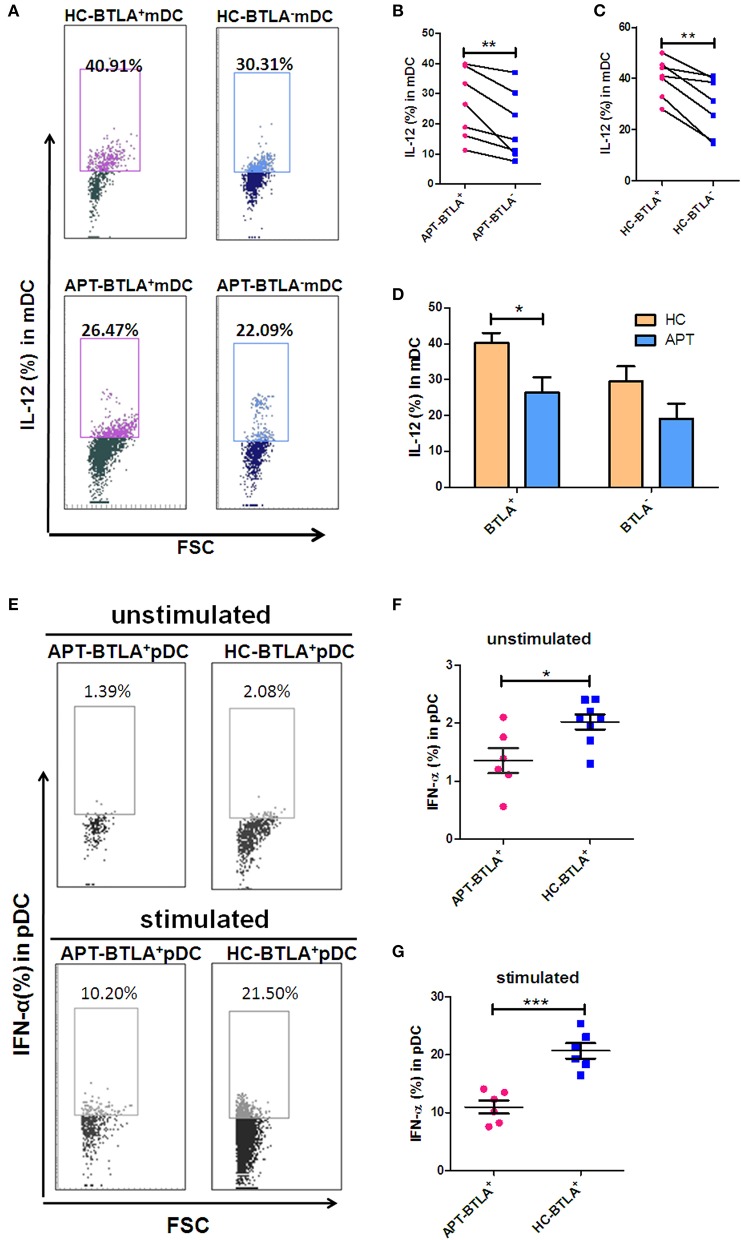
BTLA^+^ DCs in active TB showed a decreased production of IL-12 and IFN-α compared to those in HCs. As IL-12 is the dominant cytokine-inducing Th1 differentiation produced by mDCs, meanwhile IFN-α is the dominant cytokine produced by pDCs, we sought to investigate whether BTLA expression affects IL-12 and IFN-α production in mDC and pDC subsets, respectively. **(A)** Representative histograms for intracellular cytokine staining by flow cytometry analysis of IL-12 produced in BTLA^+^ or BTLA^−^ mDCs. **(B,C)** show the mean frequencies of IL-12-producing mDCs in BTLA^+^ DCs and BTLA^−^ DCs from APT patients (*n* = 6) and HCs (*n* = 6), respectively. **(D)** shows IL-12-producing mDCs in BTLA^+^ DCs and BTLA^−^ mDCs between HC and APT patients. **(E)** Representative histograms for intracellular cytokine staining by flow cytometry analysis of IFN-α produced in BTLA^+^ pDCs. **(F,G)** show the IFN-α-producing pDCs in BTLA^+^ pDCs between HCs (*n* = 6) and APT patients (*n* = 6). The *P*-values are shown in each column (ANOVA with the Bonferroni *post-hoc* test). ^*^P < 0.05, ^**^P < 0.01, ^***^P < 0.001.

We sought to examine the production of IL-12 and IFN-α in mDCs and pDCs, respectively. Although BTLA^+^ mDCs produced more IL-12 than BTLA^−^ mDCs in both HCs and APT patients (Figures 8A–C), BTLA^+^ mDCs from APT patients produced much lower IL-12 levels than those from HCs (Figures 8A,D). Moreover, direct intracellular IFN-α staining showed that APT patients showed significantly lower frequencies of IFN-α^+^ pDCs than did HCs, despite that both APT patients and HCs exhibited fewer pDCs producing IFN-α in blood (Figures 8E,F). Intriguingly, staining after 24 h *ex vivo* simulation with Mtb lysate showed that about 20% of pDCs from HCs produced IFN-α, whereas only about 10% of pDCs from APT patients produced IFN-α (Figures 8E,G). These results suggest that BTLA expression in DCs of active TB patients leads to reduced production of IL-12 and IFN-α.

### BTLA^+^ DCs in Active TB Show an Increased Production of IL-4 and TGF-β in Patients With TB

**Figure 9:**
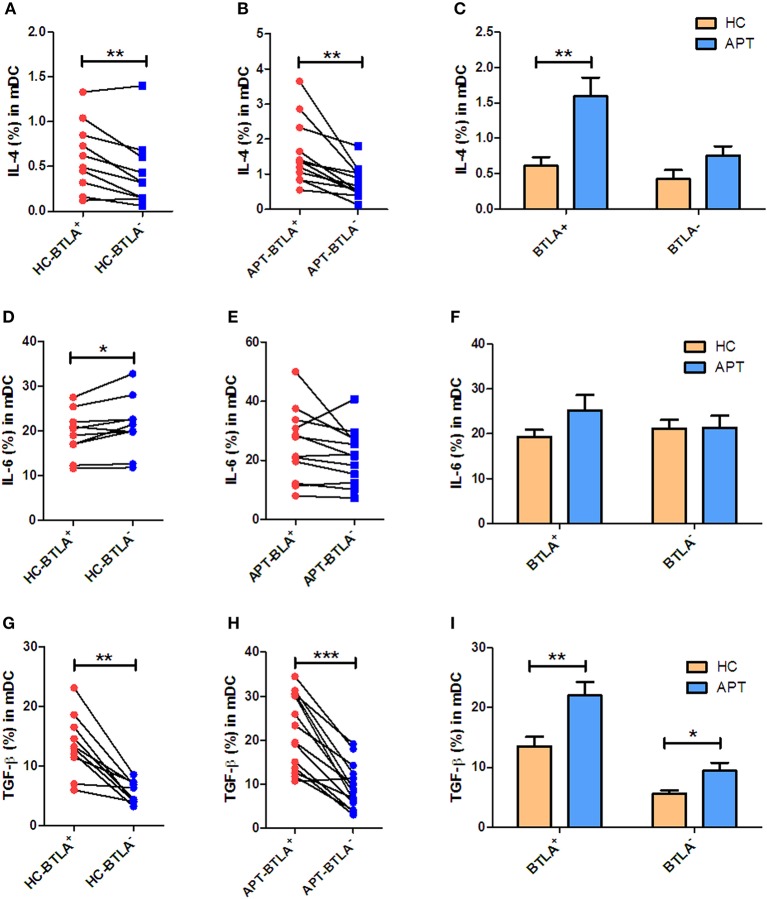
BTLA^+^ mDCs in active TB show an increased production of IL-4 and TGF-β compared with those from HCs. As IL-4, IL-6, and TGF-β produced by mDCs affect the polarization of Th cells, we sought to investigate whether BTLA expression affects IL-4, IL-6, and TGF-β production in mDCs. **(A,D,G)** show the mean frequencies of IL-4, IL-6, and TGF-β producing mDCs in BTLA^+^ and BTLA^−^ DCs from HCs (*n* = 10). **(B,E,H)** show the mean frequencies of IL-4, IL-6, and TGF-β producing mDCs in BTLA^+^ and BTLA^−^ DCs from TB patients (*n* = 12 or 14). **(C,F,I)** show the comparison of IL-4, IL-6, and TGF-β producing mDCs in BTLA^+^ and BTLA^−^ mDCs between HC and APT patients, respectively. The P values are shown in each column (Student *t*-test). ^*^P < 0.05, ^**^P < 0.01, ^***^P < 0.001.

We sought to examine the production of IL-4, IL-6, and TGF-β in mDCs. Interestingly, direct ICS showed that BTLA^+^ mDCs produced larger amounts of IL-4 and TGF-β than BTLA^−^ mDCs in both HCs and APT patients (Figures 9A,B,G,H). On the other hand, the frequency of IL-6-positive cells in BTLA^+^ mDCs in HCs was lower than that in BTLA^−^ mDCs (Figure 9D), but there was no difference in IL-6-producing mDCs in TB patients between BTLA^+^ mDCs and BTLA^−^ DCs (Figure 9E). Intriguingly, direct ICS also showed that APT patients showed significantly higher frequencies of IL-4^+^ mDCs and TGF-β^+^ mDCs in BTLA^+^ mDCs (Figures 9C,I). Moreover, APT patients showed significantly higher frequencies of TGF-β^+^ mDC in BTLA^−^ mDCs (Figure 9I), but there was no difference in the frequency of IL-6^+^ cells in BTLA^+^/BTLA^−^ mDCs between the APT and HC groups (Figure 9F). These results suggest that BTLA expression in DCs of active TB patients induces the production of IL-4 and TGF-β.

### BTLA^+^ DCs and BTLA^−^ DCs in Active TB Patients Exhibit a Reduced Capacity to Induce Allogeneic T Cell Proliferation

**Figure 10:**
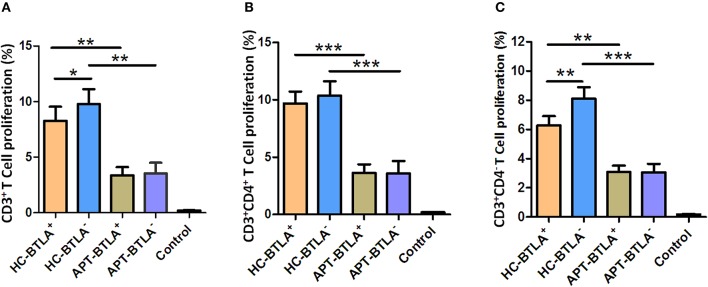
BTLA^+^ DCs and BTLA^−^ DCs in active TB patients exhibited a reduced capacity to induce allogeneic T cell proliferation. BTLA^+^ DCs and BTLA^−^ DCs were sorted by flow cytometry from sputum smear-positive APT patients (*n* = 8) and HCs (*n* = 8). Moreover, CD3^+^ T cells were isolated from an allogeneic donor and labeled with CFSE. Allo-MLC was performed by mixing BTLA^+^ DCs or BTLA^−^ DCs with allogeneic CD3^+^ T cell subsets, and T cell proliferation was determined by flow cytometry. **(A)** shows the flow cytometric histogram of the proliferation of CD3^+^ T cells stimulated by BTLA^+^ DCs and BTLA^−^ DCs. **(B,C)** show T cell proliferation in CD3^+^CD4^+^ T cells **(B)** and CD3^+^CD4^−^ T cells **(C)** stimulated by BTLA^+^ DCs and BTLA^−^ DCs from the HC and APT groups, respectively. The *P*-values are shown in each column (ANOVA with the Dunnett *post-hoc* test). ^*^P < 0.05, ^**^P < 0.01, ^***^P < 0.001. In **(A)**, *P* < 0.001 for HC-BTLA^+^ and HC-BTLA^−^ vs. Control, but *P* > 0.05 for APT-BTLA^+^ and APT-BTLA^−^ vs. Control. In **(B)**, *P* ≤ 0.05 for all HC and APT groups vs. Control. For **(C)**, *P* < 0.01 for all HC and APT groups vs. Control.

The allo-stimulatory capacity of BTLA-expressing DCs was assessed by a mixed leukocyte culture (MLC) assay (23, 30). BTLA^+^ tDCs or BTLA^−^ tDCs were sorted from the peripheral blood of HCs and active TB patients by flow cytometry. CD3^+^ T-cell subsets were isolated from allogeneic donors and labeled with CFSE. The MLC was performed by mixing BTLA^+^ tDCs or BTLA^−^ tDCs with allogeneic CD3^+^, CD3^+^CD4^+^, and CD3^+^CD4^−^ T cells, respectively, for T cell proliferation using CSFE-based flow cytometry. Interestingly, BTLA^+^ DCs and BTLA^−^ DCs in active TB patients exhibited a reduced capacity to induce allogeneic proliferative responses of CD3^+^ T cells and CD3^+^CD4^+^ T, and CD3^+^CD4^−^ T cell subsets in the MLC assay compared with those in HCs (Figures 10A–C). Notably, BTLA^+^ DCs in APT patients showed a much lower ability to stimulate the proliferation of CD3^+^ T cells and CD3^+^CD4^−^ T cells than did BTLA^−^ DCs in HCs (Figures 10A,C), but no difference was seen between BTLA^+^ DCs and BTLA^−^ DCs in APT patients (Figures 10A–C).

### BTLA^+^ DCs From Patients With Active TB Show High Polarization Capacity for Tregs and Th2, but Low Capacity for Th17 and Th22

**Figure 11:**
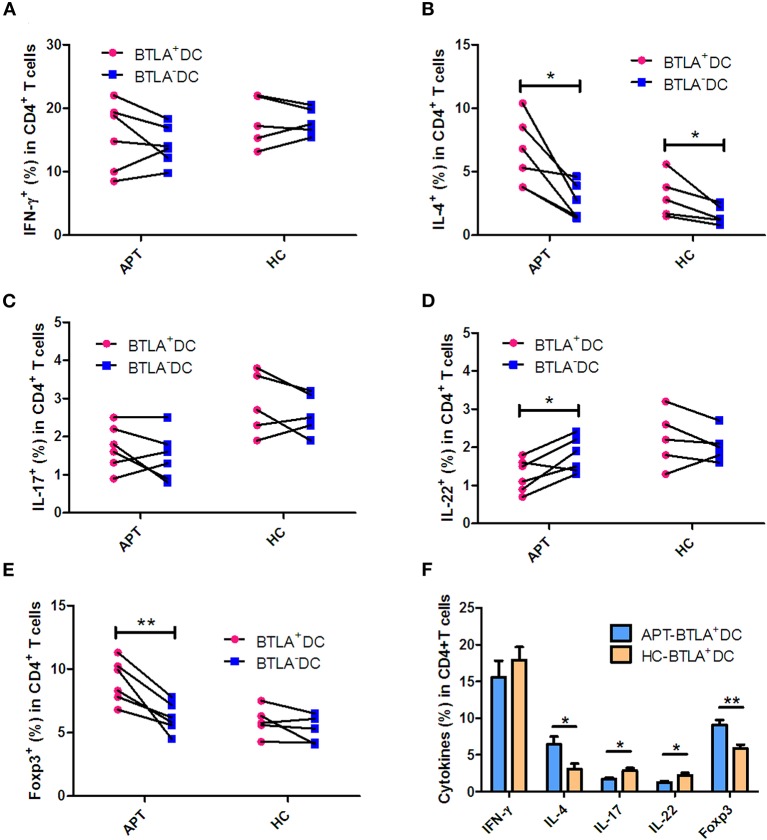
BTLA^+^ DCs in active TB patients were more readily polarized to Treg and Th2 in HCs but limited the Th17 and Th22 effector functions. BTLA^+^ DCs and BTLA^−^ DCs were sorted by flow cytometry, and host T cells were sorted by immune magnetic beads. BTLA^+^ DCs or BTLA^−^ DCs were co-cultured for 5 days with autologous T cells in the presence or absence of Mtb lysate antigen. On day 5, CD4^+^ T cell effector subsets were determined by intracellular cytokine staining. **(A–E)** show the comparison of frequencies of CD4^+^ T-cell effector subsets, Th1, Th2, Th17, Th22, and Treg, respectively, in APT (left) and HC (right) groups. **(F)** The graph data showing the frequencies of CD4^+^ T cell effector subsets, Th1, Th2, Th17, Th22, and Treg, respectively, in the co-cultures stimulated by Mtb lysate and autologous BTLA^+^ DCs from APT and HCs. The *P*-values are shown in each column (Student *t*-test). ^*^P < 0.05, ^**^P < 0.01.

DCs are the most efficient APCs and can activate and polarize the T-cell subsets. Comparative studies of the ability of BTLA^+^DCs and BTLA^−^DCs to stimulate T cell polarization have not yet been reported. Herein, we employed an auto-MLC assay, stimulated by Mtb antigen combined with intracellular cytokine staining with flow cytometry analysis to determine whether BTLA expression in DCs leads to a reduction in this function. Interestingly, BTLA^+^ DCs from APT patients exhibited a lower ability to differentiate naïve T cells into Th17 and Th22 effector cells and conversely a higher capacity to induce Th2 and Treg polarization than did those from HCs (Figure 11F). Frequencies of IL-17-and IL-22-producing CD4^+^ T cell from APT patients were significantly lower than those from HCs after the immune stimulation in the co-culture of DCs plus PBMC/T cells (Figure 11F). In contrast, frequencies of IL-4- and Foxp3-expressing CD4^+^ T cells from APT patients were significantly higher than those from HCs after stimulation by autologous BTLA^+^ DCs (Figure 11F). These changes appeared to be driven by both BTLA expression and active TB, because BTLA^+^ DCs from APT patients showed a lower capacity to induce Th22 effector cells and conversely a higher ability to stimulate Th2 and Treg polarization than did BTLA^−^ DCs (Figures 11A–E). In addition, the BTLA^−^ DCs from APT patients also displayed a less stimulatory capacity to induce Th1 and Th17 than those from HCs (Figure S2). Thus, these results suggest that both BTLA^+^ and BTLA^−^ DCs from APT patients display less stimulatory capacity to induce Th1 and Th17 than HC DC.

## Discussion

The present study is an extension of our previous studies on DC profiles and BTLA expression in CD11c+ APCs in active TB patients. The findings of this study suggest that TB-driven BTLA expression in DCs impair the expression of functional DC surrogate surface markers, reduce the production of IL-12/IFN-α and increase the production of IL-4 and TGF-β. BTLA expressed DCs from active TB patients show a reduced ability to stimulate *Mtb* antigen-driven Th17 and Th22 polarization and conversely favor the differentiation of T regulatory cells (Treg) and Th2.

BTLA was highly expressed in mDCs and pDCs from APT patients compared with those from HCs, but no difference was observed in the tDCs between APT patients and HCs. This might be attributed to the presence of other DC subsets in tDCs. In the present study, tDCs were gated by flow cytometry using Lin1^−^ HLA-DR^+^. Actually, it has been reported that there is a group of CD123^−^CD11c^−^ cells among human Lin1^−^HLA-DR^+^ PBMC (31). We found that, indeed, CD123^−^CD11c^−^ increased in APT patients (Figure S3), while the expression of BTLA in Lin1^−^HLA-DR^+^CD123^−^CD11c^−^ cells from APT patients was lower than that from HCs. Therefore, the lack of difference of BTLA expression in tDCs between APT patients and HCs may have been caused by the different expression of this molecule in the Lin1^−^HLA-DR^+^ CD123^−^CD11c^−^ cell population. Interestingly, high BTLA expression in DCs appears to be driven by active TB, as effective anti-TB therapy significantly reduces the frequency of BTLA^+^ DCs (including tDCs, mDCs, and pDCs) after 1 month of treatment in newly active TB patients. BTLA is a negative immune checkpoint molecule and, like PD-1 and CTLA-4, is expressed on activated T cells, B cells, DCs, macrophages, and some NK cells. We recently found that BTLA is highly expressed in CD11c-expressing APCs in TB patients. Herein, we extend our previous study and showed TB-driven BTLA expression and dysfunction in mDCs and pDCs. The differences in the pDCs between TB patients and HCs were small, but they were statistically significant. Regarding whether the difference is clinically significant, we agree that it is currently unknown because our global understanding of the mechanisms through which Mtb survives in a host is still insufficient. In addition, some effects are observed in BTLA^+^ and BTLA^−^ cells (e.g., the reduced ability to stimulate allogeneic T-cell proliferative responses or the less stimulatory capacity to induce Th1 and Th17 polarization), which clearly suggests that some of the effects observed in the present study are not dependent on the presence of BTLA, but on the infection by Mtb. Additional studies are still necessary to discriminate the two types of effects.

Our data suggest that BTLA expression impacts DC maturation and their antigen uptake and presentation. Before migration into the bloodstream, pDCs complete the last step of maturation in the bone marrow. On the other hand, mDCs differentiate under the influence of a combination of stimuli, including that from GM-CSF, TNF-α, and IL-4. Interestingly, the present study showed a high expression of the DC maturation markers CD83 and CCR7 in BTLA^+^ DCs, including tDCs and mDCs. The comparison between BTLA^+^ DCs and BTLA^−^ DCs by flow cytometry showed that the expression of the antigen-presenting molecule HLA-DR was much lower in BTLA^+^ tDCs from both TB patients and HCs, but was higher in mDCs. Moreover, the antigen uptake capacity was lower in BTLA^+^ DCs than in the BTLA^−^ DCs in both active TB and HCs. This is consistent with the observation that BTLA^+^ DCs had higher expression of CD83 and CCR7, which are associated with decreased antigen uptake. Combined with the observation that the BTLA^+^ DCs express lower levels antigen-presenting molecules, those results suggest a weaker function of DCs in patients with TB. Intriguingly, the expression of the co-stimulatory molecules CD80 and CD86 showed an opposite phenomenon, displaying higher CD80 expression in BTLA^+^ tDCs and mDCs. Conversely, CD86 expression was lower in BTLA^+^ tDCs and mDCs than that in BTLA^−^ DCs. CD80 has many roles in the immune system and its exact role in TB remains to be determined. The CD80-CTLA-4 interaction is known to promote the suppressive effects of Tregs (32, 33) and to induce immune tolerance (34). On the other hand, CD86 provides signal for T cell activation and survival, and decreased CD86 levels could also be involved in immune tolerance (35). CD80 and CD86 are known to be involved in complicated immune processes because they can either stimulate or inhibit the immune response depending upon the exact context of their expression (32, 36) and additional studies are necessary to unravel their exact roles in TB. Nevertheless, the results are supported by those of Sun et al. (17), who showed that BTLA is involved in an impaired response in the early stages of the immune response against listeriosis.

The present study indicated that the changes on surface markers were different between pDCs and mDCs. mDCs express many lectins, TLRs, and pattern recognition receptors for antigen uptake and presentation, and are good stimulators of naïve T cells. On the other hand, pDCs are reported to play roles in immune tolerance (11). Those two DC subtypes are clearly associated with chronic and latent Mtb infection, but additional studies are necessary to determine the exact mechanisms. Furthermore, those two subsets can be further subdivided (11), and those other subtypes were not assessed in the present study.

The stimulatory capacity of DCs, including BTLA^+^ tDCs and BTLA^−^ tDCs, from TB patients to allogenic CD3^+^ T cells, CD3^+^CD4^+^ T cells, and CD3^+^CD4^−^ T cells was much lower than that of DCs from HCs. Intriguingly, the stimulatory capacity of BTLA^+^ DCs to CD3^+^ T cell and CD3^+^CD4^−^ T cell was much lower than that of BTLA^−^ DCs in HCs, but no difference was observed between BTLA^+^ DCs and BTLA^−^ DCs in TB patients. A previous study showed that BTLA expression on CD8α^+^ DCs suppresses the responses of primary and memory CD8 T cells against *Listeria* (37). Our recent study showed that the stimulatory capacity to induce allogeneic T cell proliferation was decreased in TB patients (26).

In the present study, BTLA^+^ mDCs and pDC in TB patients exhibited a reduced ability to produce IL-12 and IFN-α, as well as an increased ability to produce IL-4 and TGF-β, compared with those from HCs. IL-12 and IL-4 are key cytokines in determining activation, differentiation, proliferation, and survival of T cells (38–40). TGF-β plays crucial roles in the development of conventional, regulatory, and innate-like T cells (41). Although our DC plus T cell assay did not uncover impaired Th1 responses in TB patients, the reduced IL-12/IFN-α production by DCs in active TB suggests that BTLA^+^ DCs of TB patients have potential dysfunction in initiating and sustaining adaptive T-cell immune responses. Intriguingly, in the autologous MLC assay post-stimulation by the Mtb antigen, BTLA expression in DCs in active TB was found to support T cell differentiation into Th2 and Foxp3^+^ Treg, but less into anti-TB effector T cell subsets such as Th17 and Th22. IL-4 participate in the differentiation of T cells into Th2 cells (38). IL-4 and TGF-β can modify the phenotype of CD4^+^ T cells, and these cells can then produce large amounts of IL-9, which is a recently described type of effector T cells (Th9 cells) (38). Th9 cells have been shown to be increased in the pleural effusion of patients with APT, but the exact role of those cells in TB remains to be determined (42). Unfortunately, Th9 cells were not examined in the present study. Furthermore, the roles of IL-4 in the immune system are complex and far from being well-understood (38), and the balance between IL-4 and TGF-β determines the establishment of immune tolerance or response (43). Since both IL-4 and TGF-β were increased in BTLA^+^ mDCs and pDC from TB patients, their exact impact on the response to TB remains to be elucidated.

A previous study reported that efficient induction of Tregs was directed by BTLA^+^ DEC205^+^ CD8^+^ CD11c^+^ DCs. In contrast, T cell activation in a steady state by total CD11c^+^ BTLA^−^ DCs did not induce Treg cells and did not exert a lasting impact on subsequent immune responses (18). Interestingly, decreased BTLA expression is associated with increased Th17 and Th1 responses and the activation of BTLA inhibits abnormal Th17 and Th1 responses and IL-22 expression in both patients and controls (20). These data appear to be supporting the present study.

The present study has limitations. The results were obtained from a small number of TB patients and HCs. Only two subsets of DCs were examined and the changes in the various immune cells were not assessed. In addition, cytokines and immune effectors/regulators were not measured in the blood of the subjects. It is important to highlight the fact that the present study was only designed to detect associations, not causal relationships. HVEM and BTLA are involved together in a pathway involved in T cell activation (44), but HVEM was not examined in the present study. Finally, the T cell populations were not examined in response to BTLA status. A previous study by our group showed that BTLA expression on CD4^+^ and CD8^+^ T cells was associated with TB progression (45). Additional studies are necessary to address those issues.

In summary, the present study suggests that active TB drives BTLA expression in DCs, affecting their biological characteristics and immune functions. In this context, TB-driven BTLA expression appears to be associated with decreased capacities of DCs to produce the key cytokine IL-12 and to induce T cell proliferation and to differentiate into Th subsets, and increased capacities to produce IL-4 and TGF-β. Ultimately, such aspects of DC dysfunction will alter anti-TB immune responses and immunity. Our findings support further additional mechanistic studies for the elucidation of the underlying mechanisms.

## Data Availability Statement

The raw data supporting the conclusions of this article will be made available by the authors, without undue reservation, to any qualified researcher.

## Ethics Statement

The study was approved by the Internal Review and the Ethics Boards of Guangdong Medical University and Dongguan Sixth People's Hospital, and informed consent in writing was obtained from all the study subjects or parents/guardians on behalf of all child participants.

## Author Contributions

J-FX conceptualized and designed the study. Y-BL, J-AZ, W-DW, and CC performed experiments. Y-BL, J-AZ, W-DW, LS, H-LL, and HX analyzed data. G-BL, YP, HL, G-XH, D-DW, L-LY, and B-YZ performed clinical investigation, J-FX and W-DW drafted the initial manuscript. J-FX and ZC reviewed and revised the manuscript. All authors approved the final manuscript as submitted and agreed to be accountable for all aspects of the work.

### Conflict of Interest

The authors declare that the research was conducted in the absence of any commercial or financial relationships that could be construed as a potential conflict of interest.

## Supplementary Material

Figure S1 Flow cytometry gate strategy. Peripheral blood mononuclear cells (PBMCs) were gated on FSC and SSC dot plots. Total dendritic cells (DCs) were gated from PBMCs by HLA-DR expression and Lin1 negativity. The DC subsets were gated as: CD11c^+^CD123^−^ for mDCs and CD11c^−^CD123^+^ for pDCs. The expression of BTLA, DC maturation markers CD83 and CCR7, co-stimulatory molecules CD80 and CD86, and antigen-presenting molecule HLA-DR was analyzed in mDCs and pDCs.

Figure S2 Stimulatory function of BTLA^−^ DCs to naïve T cell polarization. BTLA^+^ tDCs and BTLA^−^ tDCs were sorted by flow cytometry. Naïve T cells were sorted by immune beads. BTLA^+^ tDCs or BTLA^−^ tDCs were co-cultured with autologous naïve T cells, and Mtb lysis solution was added as a stimulator to the culture system. After 5 days, CD4^+^ T cell subsets were determined by intracellular cytokine staining. The comparative analysis of the stimulatory function of BTLA^−^ DCs between APT patients and HCs is shown. The *P*-values are shown in each column. ^*^*P* < 0.05.

Figure S3 Expression of BTLA in Lin1^−^HLA-DR^+^CD123^−^CD11c^−^_cells. Expression of BTLA in Lin1^−^HLA-DR^+^CD123^−^CD11c^−^ cells in PBMCs both in HCs and in APT patients was analyzed by flow cytometry. Flow cytometry gate strategy is showing Figure S1. The expression of BTLA in Lin1^−^HLA-DR^+^CD123^−^CD11c^−^ cells is showing in the Figure.

Table S1 Clinical characteristics of the enrolled subjects.

Table S2 The clinical data of studied subjects.
