# Supplementary figures and images for: BTLA-Expressing Dendritic Cells in Patients With Tuberculosis Exhibit Reduced Production of IL-12/IFN-α and Increased Production of IL-4 and TGF-β, Favoring Th2 and Foxp3+ Treg Polarization
Source: Front Immunol. 2020 Mar 31;11:518. doi: 10.3389/fimmu.2020.00518 (PMC7136538; doi:10.3389/fimmu.2020.00518)

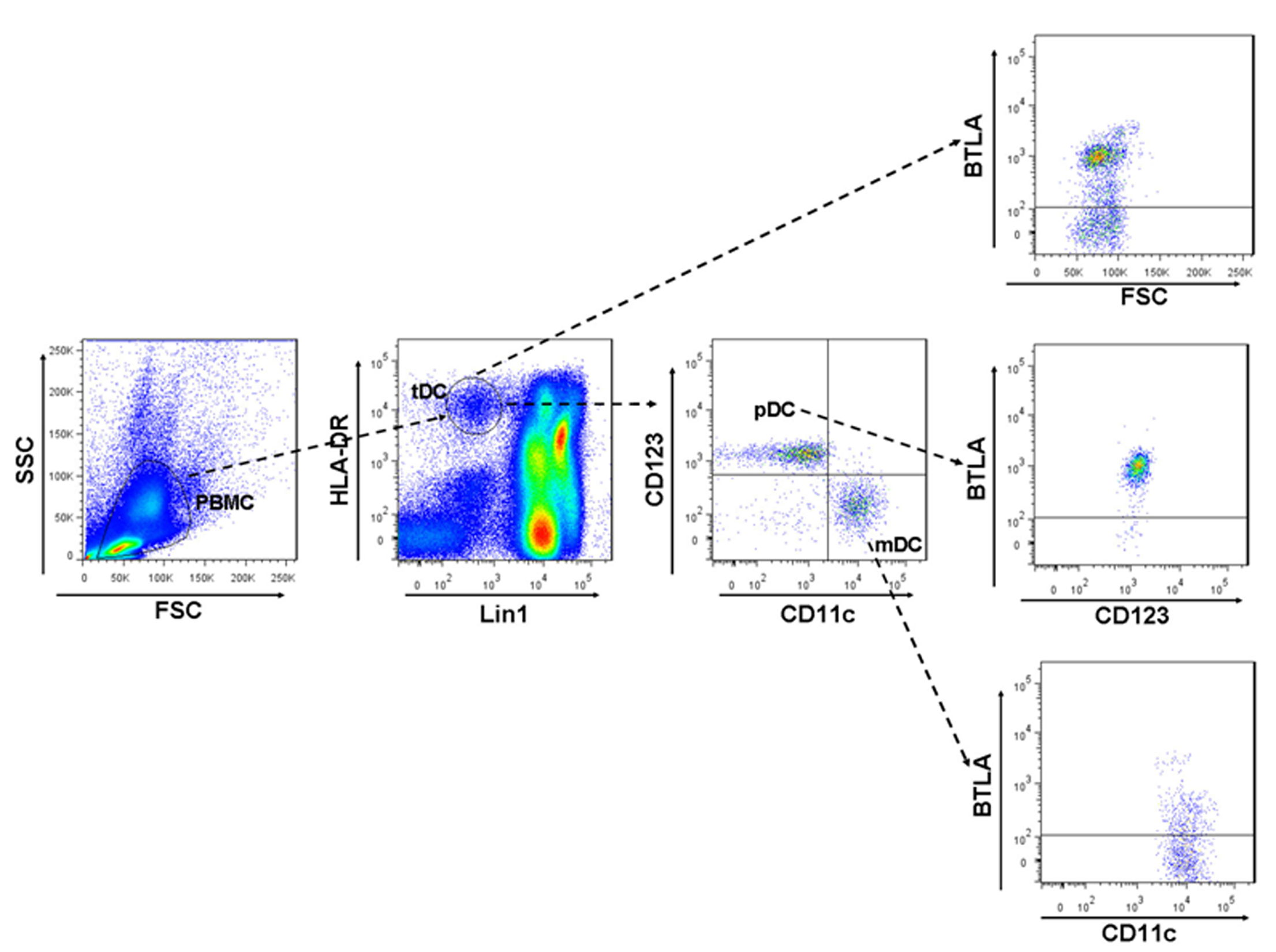

Supplement: Figure S1 — Flow cytometry gate strategy. Peripheral blood mononuclear cells (PBMCs) were gated on FSC and SSC dot plots. Total dendritic cells (DCs) were gated from PBMCs by HLA-DR expression and Lin1 negativity. The DC subsets were gated as: CD11c+CD123− for mDCs and CD11c−CD123+ for pDCs. The expression of BTLA, DC maturation markers CD83 and CCR7, co-stimulatory molecules CD80 and CD86, and antigen-presenting molecule HLA-DR was analyzed in mDCs and pDCs. [file Image_1.TIF]

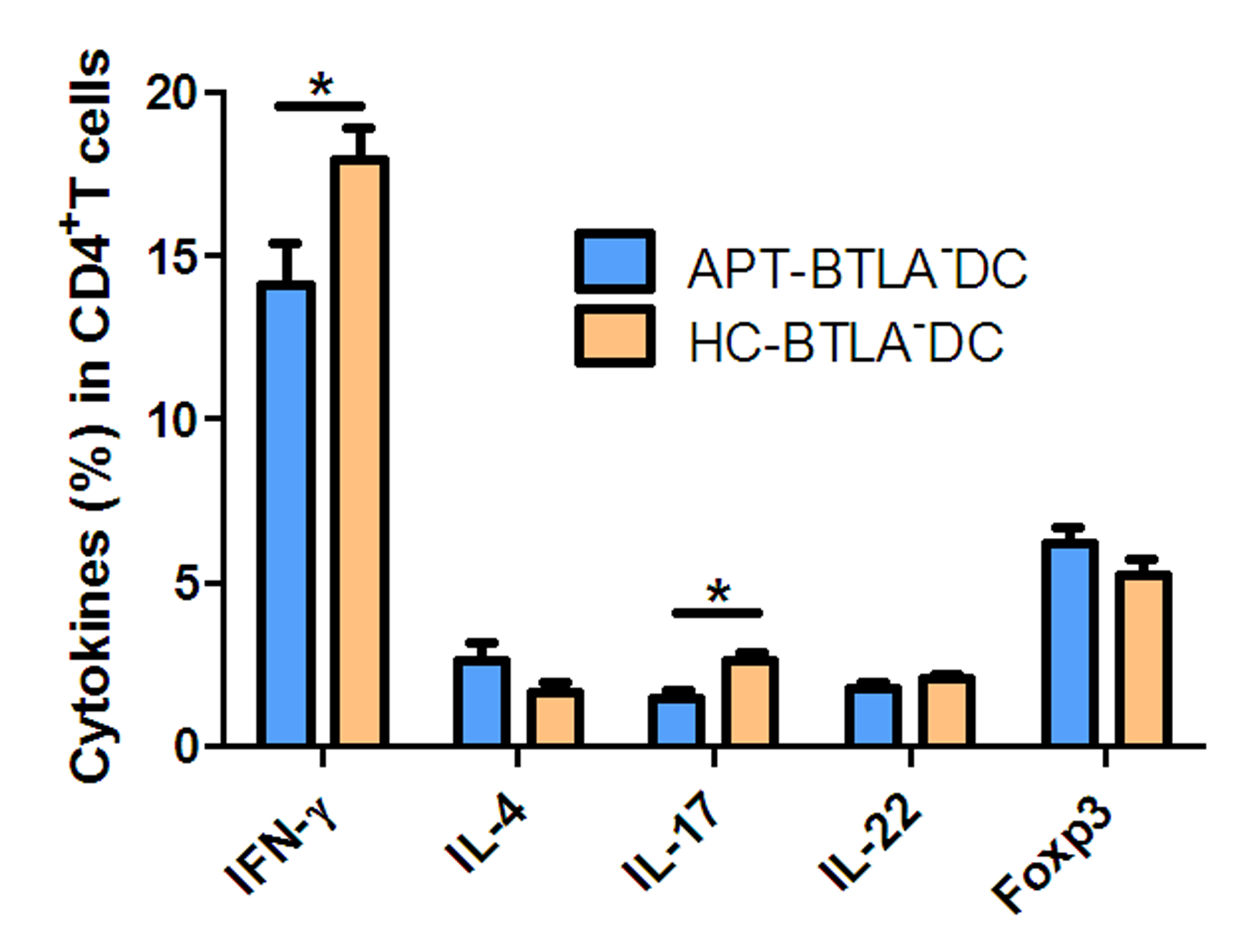

Supplement: Figure S2 — Stimulatory function of BTLA− DCs to naïve T cell polarization. BTLA+ tDCs and BTLA− tDCs were sorted by flow cytometry. Naïve T cells were sorted by immune beads. BTLA+ tDCs or BTLA− tDCs were co-cultured with autologous naïve T cells, and Mtb lysis solution was added as a stimulator to the culture system. After 5 days, CD4+ T cell subsets were determined by intracellular cytokine staining. The comparative analysis of the stimulatory function of BTLA− DCs between APT patients and HCs is shown. The P-values are shown in each column. *P < 0.05. [file Image_2.TIF]

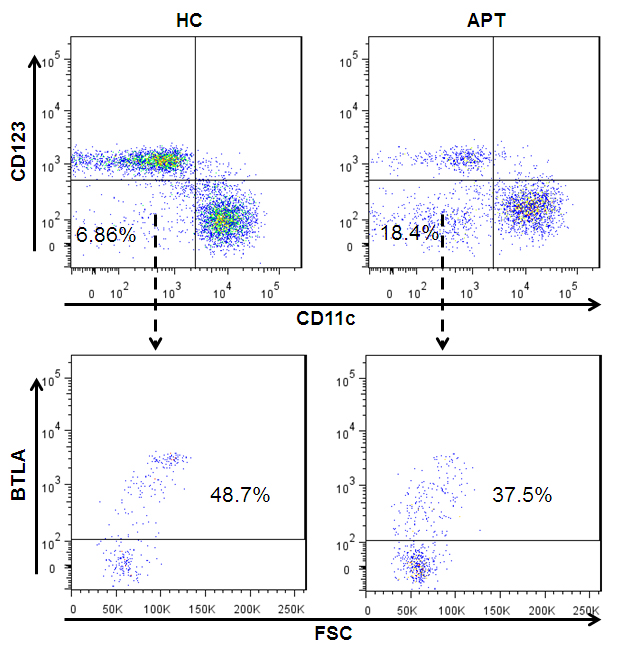

Supplement: Figure S3 — Expression of BTLA in Lin1−HLA-DR+CD123−CD11c−_cells. Expression of BTLA in Lin1−HLA-DR+CD123−CD11c− cells in PBMCs both in HCs and in APT patients was analyzed by flow cytometry. Flow cytometry gate strategy is showing Figure S1. The expression of BTLA in Lin1−HLA-DR+CD123−CD11c− cells is showing in the Figure. [file Image_3.TIF]
